# Calculating dissolved marine oxygen values based on an enhanced Benthic Foraminifera Oxygen Index

**DOI:** 10.1038/s41598-022-05295-8

**Published:** 2022-01-26

**Authors:** M. Kranner, M. Harzhauser, C. Beer, G. Auer, W. E. Piller

**Affiliations:** 1 Geological-Palaeontological Department, Natural History Museum Vienna, Burgring 7, 1010 Vienna, Austria; 2 Institute of Earth Sciences (Geology and Palaeontology), NAWI Graz Geocenter, University of Graz, Heinrichstr. 26, 8010 Graz, Austria; 3 PwC Austria, Donau-City-Straße 7, 122 Vienna, Austria

**Keywords:** Ecology, Biodiversity, Biooceanography, Ecosystem services, Palaeoecology, Marine biology, Palaeontology, Biogeochemistry

## Abstract

Marine oxygen minimum zones (OMZs) trap greenhouse gases, reduce livable habitats, a critical factor for these changes is the amount of dissolved oxygen (DO). The frequently used tool to reconstruct DO values, the Benthic Foraminifera Oxygen Index (BFOI), showed major shortcomings and lacks effectiveness. Therefore, we enhanced the BFOI and introduce enhanced BFOI (EBFOI) formulas by using all available data benthic foraminifers provide, calculating the whole livable habitat of benthic foraminifers, including bottom water oxygenation (BWO) and pore water oxygenation (PWO). Further, we introduce for the first time a transfer function to convert EBFOI vales directly into DO values, increasing efficiency by up to 38%. All formulas are calibrated on modern samples and applied to fossil datasets. Our new approach provides a major improvement in defining and reconstructing marine oxygen levels and eutrophication, by, providing a new toolset for understanding past changes and tracking actual and predicted future expanding OMZs.

## Introduction

The ocean covers about 97% of the Earth's physical habitat space^1^. Therefore, changes in ocean chemistry and biodiversity and their faster response to physical and atmospheric changes also affect the terrestrial realm and should be observed closely^2^. Hence, dissolved oxygen (DO) values gained attention over the last decades, not only for understanding the ecology but also as an important factor for the interdependence with ocean circulation, climate, and evolution of marine life. Especially linking human triggered changes (like pollution) in ocean chemistry led to an increase in scientific and public interest with the main focus on studying low oxic water columns, leading to a general reduction of livable habitat greatly affecting marine biota^2–7^. Areas with low DO values lead to the development of so-called Oxygen Minimum Zones (OMZs), resulting in a severe reduction of biodiversity of macro- and micro-organisms^3,7–11^. Changes leading to a depletion of oxygen in the world oceans represent a major interference of chemical processes, marking OMZs as one of the major drivers in the oceanic metabolic cycle^1,10–14^. OMZs are responsible for a loss of around 20–40% of oceanic nitrogen and lead to the production of nitrous oxide, showing the severe impact on the nitrogen cycle^1,14–16^, and the effect on the carbon cycle gives an even bigger impact of OMZs by releasing carbon dioxide to the atmosphere and serving as methane reservoir^12,13,17^. With expanding OMZs, methane accumulations move closer to zones of atmospheric exchange, drastically speeding up global warming by releasing this potent greenhouse gas^18–23^. This process emphasizes the importance of dissolved oxygen as one of the most important environmental variables, and reconstructing changes of oxygen conditions for recent and geological times garnered much interest (Refs.^24–44^). Although hypoxic and anoxic environments have existed through geological time, the recent increase of hypoxia and anoxia throughout all world oceans, especially in coastal areas, is alarming^1,5,11,46,47^. Usually, hypoxia and anoxia, accompanied by severe reduction of benthic life, is reported only seasonally, followed by a re-establishment of normal oxic conditions, but this re-establishment is now diminishing^48–50^. Instrumental records since 1960 show that DO values have been decreasing drastically throughout the last decades and are presumed to drop even further in the coming decades^1,5^. From the 1960s, 45 different areas representing low oxic conditions were reported. That number increased considerably up to 700 different locations in 2011. Low oxic conditions now even persist in areas which used to be considered as oxygen stressed^1,5^. These developments and the occurrence of low oxic conditions in already shallow coastal environments point to a rapid expansion of OMZs^1,9,13,14,21,22,46,48,49^.

The last, globally widespread OMZs culminated in ocean-wide anoxia, recorded as the Oceanic Anoxic Events (OAE) of the Mesozoic. For the development of the OAEs two major drivers were deduced: (1) The Late Cretaceous transgression leading to shallower epicontinental and marginal seas with an increase in primary production. (2) The existence of a relatively warm global climate, reducing the supply of cold oxygenated bottom water masses to the world oceans through the global thermohaline circulation^51,52^.

Recent observations of the rising global sea level^53–55^ and the above-described spreading of OMZs are reminiscent of the conditions leading to the OAEs of the Mesozoic. The depletion of oxic conditions may be a premonition of an even more significant future change in global climate, reduction of biodiversity, and accompanying extinction events, which proves the observation of DO values to be indispensable for modern science and environmental protection.

Within recent oceans, DO values can be measured with modern equipment, but these techniques still need improvement^28,29,33^. The enhancement and optimization of DO measurements are time-consuming, expensive, and simply impossible for the comprehensive dataset that would be needed to track and analyze oxic conditions. A better and easier way to track oxic levels on the seafloor would be the application of proxy data. Using modern analogue based proxy calibrations by comparing them to measured DO values enabled detailed reconstructions of past marine oxygen conditions and was even successfully applied for the fast assessment of modern environmental changes. Estimations of DO values have been based on several different proxy parameters, including isotope analyses^56–58^, rare earth element concentrations^59^ and intensity of bioturbation^24,26^.

Nevertheless, analyses on benthic foraminifers proved to be the preferred method to estimate oxygen levels^28,29,33,35,37–39,43,44,60–65^. As indicated above, benthic organisms are the first to be influenced and most impaired by changes in dissolved oxygen due to their limited mobility, making them the preferred proxy archive. Kaiho introduced the Benthic Foraminifers Oxygen Index (BFOI) in 1991 as a powerful tool to estimate changes in bottom-water oxygenation (BWO) for geological records and also published the first calibrations on recent datasets in 1994^28,29^. Since then, many authors have reconstructed oxic conditions with fossil datasets of different timeframes, partly using these BFOI calculations^33,34,66–69^. Kaminski also tested the BFOI for Mediterranean water masses using 30 samples from the Marmara Sea^33^.

However, the detailed analyses of hundreds of Miocene samples of the Austrian Vienna Basin^70,71^ revealed major inconsistencies between calculated BFOI values^29^ and the relative trends of oxygen values^71^ based on benthic foraminifers. Therefore, we saw a critical need to reevaluate the original calibrations of Kaiho^29^ to further improve the usability of benthic foraminifers as a high-resolution proxy for dissolved oxygen in modern and past marine settings.

The original formula of Kaiho^29^ is solely based on calcareous benthic foraminifers and strictly distinguishes between samples containing oxic indicators and samples lacking oxic indicators. If oxic indicators are present, Kaiho postulated the following formula:

1 $$BFOI=100\left(\frac{O}{\left(O+D\right)}\right).$$

This formula gives values between > 0 and 100, suggesting that the BWO is at least low oxic as soon as any oxic indicator is present in the sample. If no oxic indicators are present, a second formula is used:

2 $$BFOI=50\left(\frac{S}{\left(S+D\right)}-1\right).$$

Values from 0 to − 50 can be calculated using the second formula suggesting sub- to dysoxic conditions only for samples without oxic indicators.

**Figure 1:**
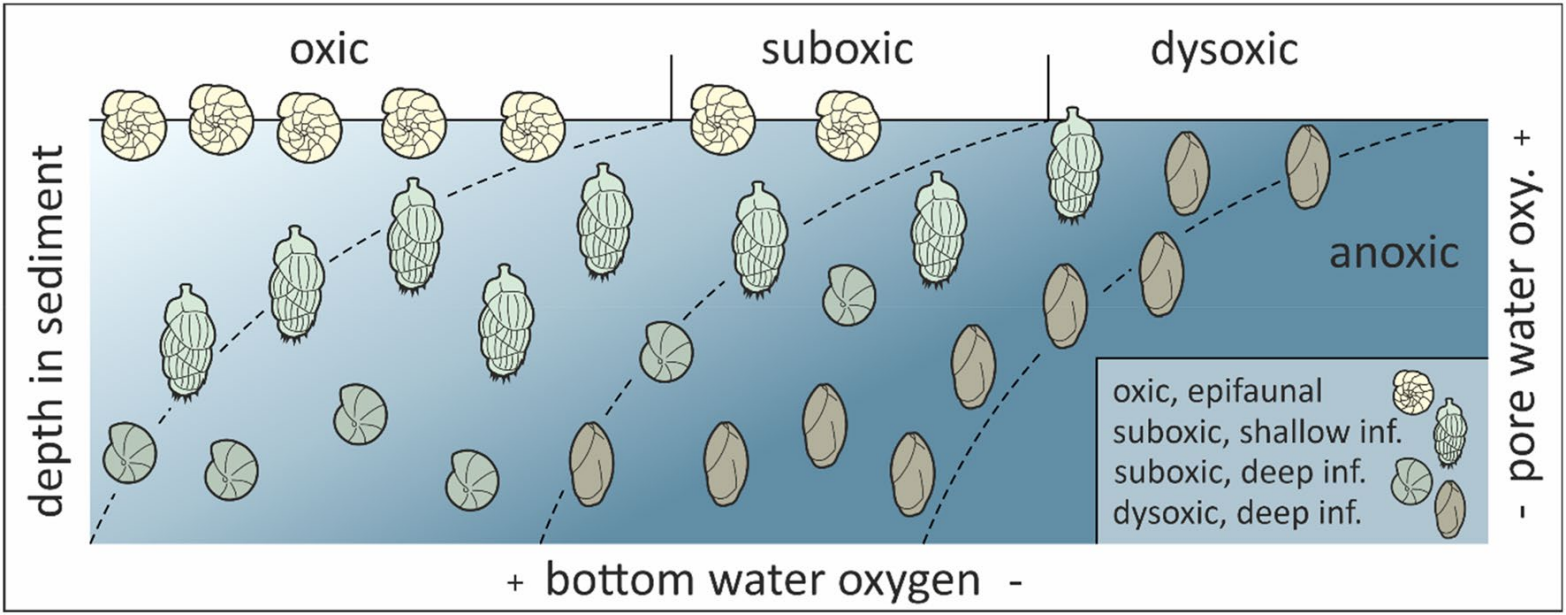
Oxygen model combining BWO and PWO. Oxygen model [(modified TROX model after Jorissen^84^; modeled after Koho^90^] combining bottom water oxygenation and pore water oxygenation using benthic foraminifers.

We realized that the formula used if oxic indicators are present lacks consideration of suboxic indicators, which leads to an overestimation of DO values. Further, sedimentary samples represent rather a combination of BWO and pore water oxygenation (PWO), especially when infaunal species are considered together with epifaunal species^29^. So, it is a general disadvantage of the BFOI that only a reconstruction of BWO was attempted, whereas reconstructing the oxygenation of the whole livable habitat, including BWO and PWO, is mandatory to use benthic foraminifers as a reliable proxy for DO levels (Fig. 1).

The BFOI uses only benthic calcareous foraminifers to reconstruct BWO. Furthermore, using only calcareous benthic foraminifers leads to a major loss of information due to the exclusion of agglutinated species. Agglutinated taxa account for 832 valid genera, about 1/3 of all valid genus-level taxa of foraminifers^72,73^. Recent molecular phylogenies suggest that agglutinated genera account for over 2/3 of all foraminiferal genera throughout the geological record^74^. Furthermore, agglutinated foraminifers are the only foraminifers that inhabit the ocean floor underneath the calcium compensation depth^75^. Therefore, agglutinated foraminifers were included in our new calculation of the BFOI, thereby utilizing the full information provided by benthic foraminiferal assemblages.

To achieve this, comprehensive revision of the formula was necessary and calibrations were conducted on the original dataset of Kaiho^29^ and seven recent datasets of Piller and Haunold^76^, Schuhmacher^30^, Kaminski^33^, Ama^77^, Charrieu^78,79^, Groenevelt^80^. Furthermore, we combined these modern datasets with additional fossil datasets of the Paleocene^81^, the Oligocene^82^ and the Miocene^71^ in order to create an enhanced BFOI (EBFOI) calculation and to provide a transfer function to convert EBFOI values to DO values [ml/l] directly.

## Results

In order to calculate (E)BFOI values, benthic foraminifers are separated into three groups: "oxic" (O; > 1,5 ml/l), "suboxic" (S; 1.5–0.3 ml/l) and "dysoxic" (D; 0.3–0.1), based on extant literature data on oxygen requirements of foraminifers (417 species-level taxa with their oxygen requirements are compiled in Appendix Table S3; for more references see publications of Kaiho^29^, Schuhmacher^30^, Kaminski^33^, Kranner^71^, Jorissen^45^, Ohkushi^36^, Moffit^42^, Palmer^40^ and Tetard^41^).

### New formulas

**Figure 2:**
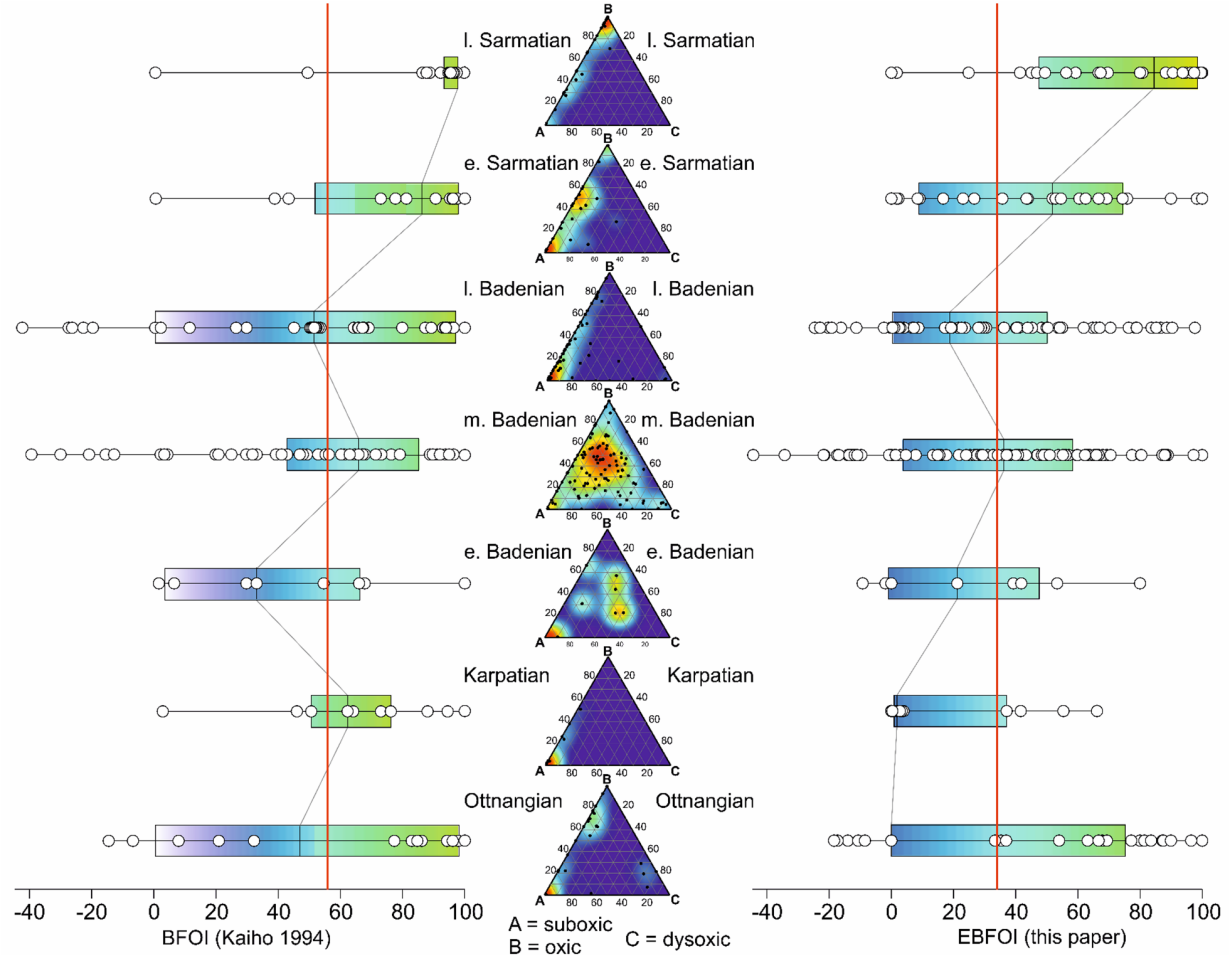
Old and new BFOI values vs. qualitative oxygen analyses. Comparing qualitative oxygen data as ternary diagrams (A = suboxic, B = oxic, C = dysoxic) with a probability density map of Kranner^71^ and the BFOI values calculated after Kaiho^29^ and our newly calculated EBFOI values (Eqs. 3–5) as boxplots. Chronostratigraphy/geochronology follows the regional stage/age concept of the Central Paratethys (Ottnangian = mid-Burdigalian, Karpatian = late Burdigalian, early and middle Badenian = Langhian, late Badenian = early Serravallian, Sarmatian = late Serravallian). Within the ternary plots, points represent the same single samples as the white dots in the boxplot diagram. Warm colors in the ternary diagrams represent a high concentration of samples and therefore a high probability of affiliation to the oxic conditions, whereas the coloring of the boxplots visualize the (E)BFOI values (green = high, blue = medium and violet = low). The red line shows the overall mean BFOI value^56^ of the BFOI values after Kaiho^29^ and the overall mean^34^ of our newly calculated EBFOI values (Eqs. 3–5).

A major offset towards overestimating oxic conditions, considerably increasing if high numbers of suboxic indicators are present within the samples, between qualitative data and calculated BFOI values was realized for the fossil datasets [e.g., the dataset of Kranner^71^; Fig. 2]. Similar patterns of mismatches were observed by checking the oxygen indications of all recent datasets and corresponding measured dissolved oxygen (mDO) values, explaining the frequent use of qualitative reconstructions and descriptive methods to estimate DO conditions of fossil datasets. We used three steps to overcome these misfits and enhance the methods introduced by Kaiho^29^.

1. Including suboxic indicators to the equation applicable when oxic indicators are present (Eq. 1). Considering the broad range of oxic conditions that suboxic foraminifera (S) can withstand, we included them with half of the weight $(\frac{S}{2}$) of oxic and dysoxic indicators.
2. The general overestimation of oxic conditions results from the assumption that conditions have to be at least low oxic if any oxic indicators are present, no matter how low abundant they are. Thus, we used the equation when no oxic indicators are present (Eq. 2) for samples with low abundances (monadic percentage of the total assemblage) of oxic indicators but further include oxic indicators to the formula to prevent underestimation of oxic conditions.
3. These two methods turned out to be good indicators for calculations of the upper and lower end of the spectrum. Nevertheless, there are many habitats in between that can only be poorly captured using these enhancements. We suggest using the arithmetic mean of both formulas if a sample contains higher amounts of dysoxic than oxic indicators (when $O\ge 10$).

Adding the suboxic indicators to the formula of Kaiho^29^ (Eq. 1) results in Eq. (3) (Eq. 3):

3 $${\text{EBFOI}} = 100\left( {\frac{O}{{\left( {O + D + \frac{S}{2}} \right)}}} \right).$$

Adding the oxic indicators to the formula of Kaiho^29^ results in Eq. (4) (Eq. 4):

4 $$EBFOI = 50\left( {\frac{S}{{\left( {S + D} \right)}} - 1} \right) + O.$$

If dysoxic indicators represent more than the oxic indicators of a sample ($$D>O)$$, the mean EBFOI of the whole livable habitat is calculated by equation (Eq. 5):

5 $$EBFOI = \frac{{100\left( {\frac{O}{{\left( {O + D + \frac{S}{2}} \right)}}} \right) + 50\left( {\frac{S}{{\left( {S + D} \right)}} - 1 + \frac{O}{2}} \right)}}{2}.$$

Thereby we enhance the BFOI method by (1) combining BWO with PWO, (2) including also agglutinated taxa, and (3) use a better assignment of oxygen requirements (e.g., small oxic taxa were considered as suboxic in previous calculations, but we prefer using general oxygen requirements) and therefore, results in giving the DO available for the whole livable habitat as described hereinafter.

Using three fixed points (100 BFOI/6 [ml/l]), (0 BFOI/1.5 [ml/l]) and (− 50 BFOI/0.1 [ml/l]) (see Table 1) to calculate the exponential formula ($a\times {e}^{b\times x}-c$) provides distinct values for each variable (a = 5.28475; b = 0.00616; c = 3.78475) leading to the following equation (Eq. 6):

**Table 1:** Relation of (E)BFOI and DO. Oxygenation concentration thresholds and relation of oxygenation and corresponding dissolved oxygen values (DO) [ml/l] to (E)BFOI values, modified after Kaiho^29^.

	DO [ml/l]	(E)BFOI
High oxic	3.0 to 6.0	50 to 100
Low oxic	1.5 to 3.0	0 to 50
Suboxic	0.3 to 1.5	− 40 to 0
Dysoxic	0.1 to 0.3	− 50 to − 40
Anoxic	0.0	− 55

6 $$DO[\frac{ml}{l}]=5.28475\times {e}^{0.00616\times x}-3.78475.$$

### Calibration with recent datasets

To calibrate our new formula, we compare the original datasets of Kaiho, as well as six other recent datasets of different settings^30,33,76–80^ with the results of the original BFOI formula (Ref.^29^; Eqs. 1–2), the EBFOI formulas (Eqs. 3–5), available measured dissolved oxygen (mDO) values and our calculated dissolved oxygen (cDO) values using Eq. (6). All raw data and a cross plot showing the correlation of the BFOI^29^ and EBFOI values are available in the supplementary material (Table S1, Fig. S1).

In summary, the used dataset includes a total of 278 recent samples, out of which 142 are associated with mDO values and the others have at least descriptive information of BWO. Only samples associated with mDO values are used to validate the new transfer function. Samples providing purely descriptive information are, in addition, used to calibrate the calculations of the EBFOI. These samples originate from different globally distributed locations and cover various depositional environments and bottom water DO values.

**Figure 3:**
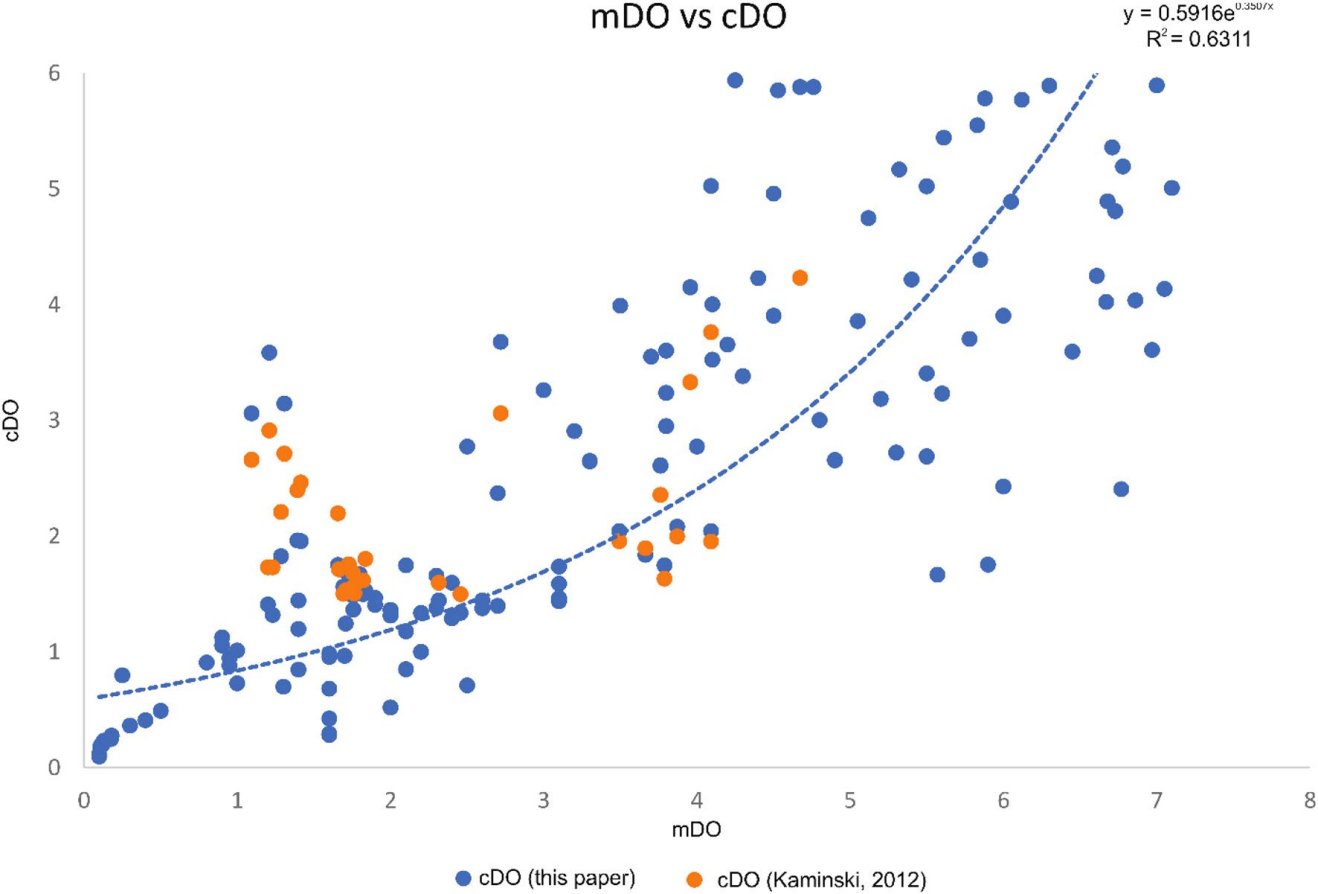
All available recent mDO vs new cDO including cDO values of Kaminski. cDO values using the EBFOI (blue dots) compared to the cDO values by Kaminski (Ref.^33^; orange dots) with an added best fit exponential trend line (R^2^ = 0.6311) showing the higher reliability of the EBFOI compared to the BFOI as a proxy for DO.

**Figure 4:**
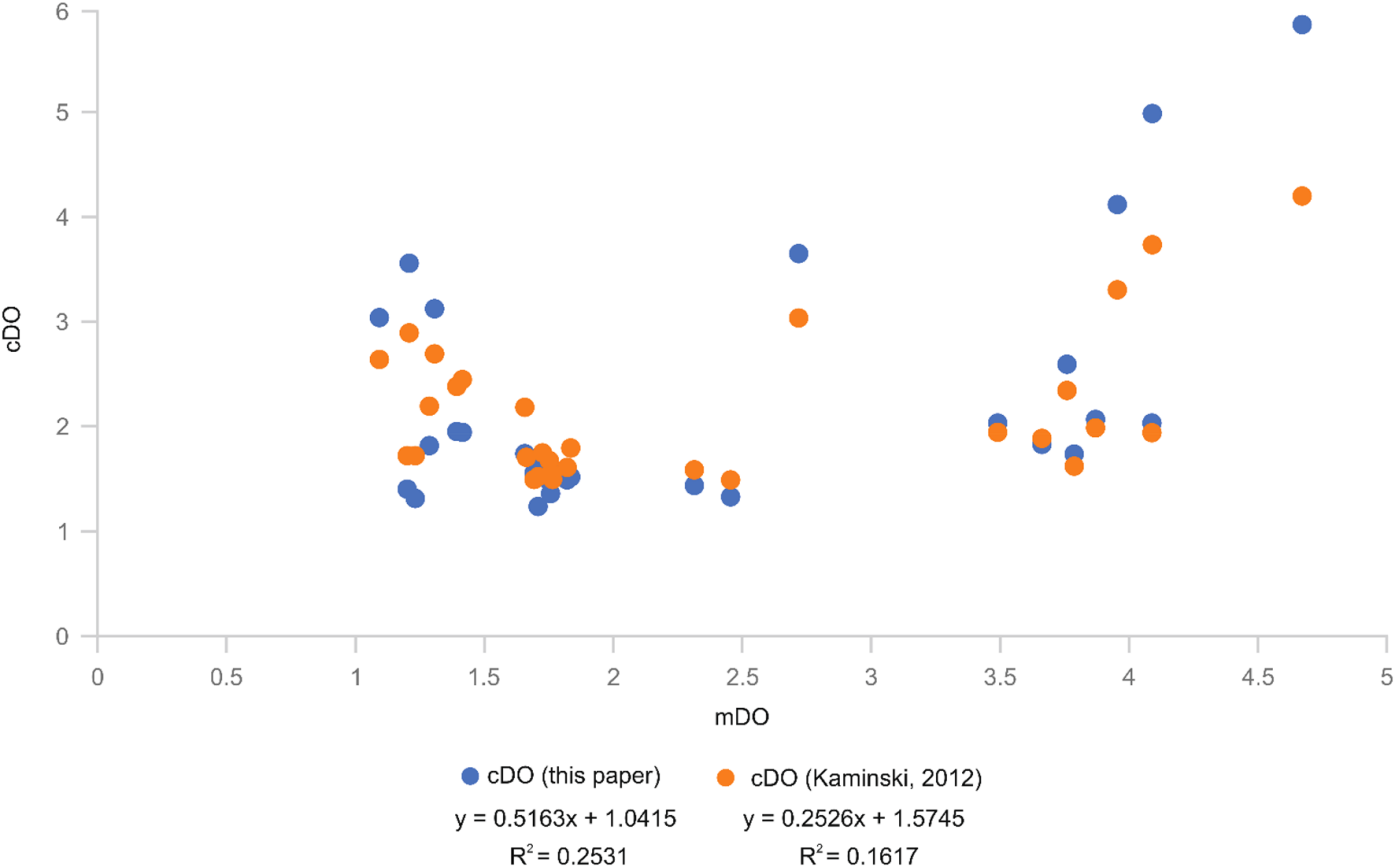
mDO vs cDO of this paper and cDO of Kaminski. Comparing the mDO values measured by Kaminski^33^ to the cDO values of Kaminski (Ref.^33^; orange dots) and cDO values of this paper (blue dots).

The original dataset of Kaiho^29^ includes 72 samples of the N. Atlantic (6), S. Atlantic (3), Indian Ocean (5), S. Pacific (7), N. Pacific (4), the Izu-Bonin area (16), the area off Onahama (14), the area off Mexico (8), the Gulf of Mexico (2), the area off California (1), the Mediterranean (4) and the Red Sea (2). The dataset (11 samples) of Schuhmacher^30^ was derived from the oxygen minimum zone of the Pakistan continental margin (Arabian Sea). Seventy-two^72^ samples from the shallow Safaga Bay of the Red Sea, investigated by Piller and Haunold^76^. Amao^77^ investigated 29 samples of high oxic deposits of the Persian Gulf and a total of 30 samples of the low oxic Baltic Sea were analyzed by Charrieau^78,79^ and Groenevelt^69^. We compared all available mDO values to our cDO values (Fig. 3) and were able to show high reliability shown with an exponential trend line (R^2^ = 0.6311) of our new calculation method of DO values. The dataset (30 samples) of Kaminski^33^ derived from generally low to high oxic deposits of the Marmara Sea also provides calculated dissolved oxygen (cDO [ml/l], Kaminski) values assuming a linear regression resulting of the BFOI values. We compared the cDO values of Kaminski^33^ to our new cDO values (Fig. 4) and were able to show that our new formula results in a higher R^2^ value (an increase of 9.1%) than the cDO values of Kaminski.

**Figure 5:**
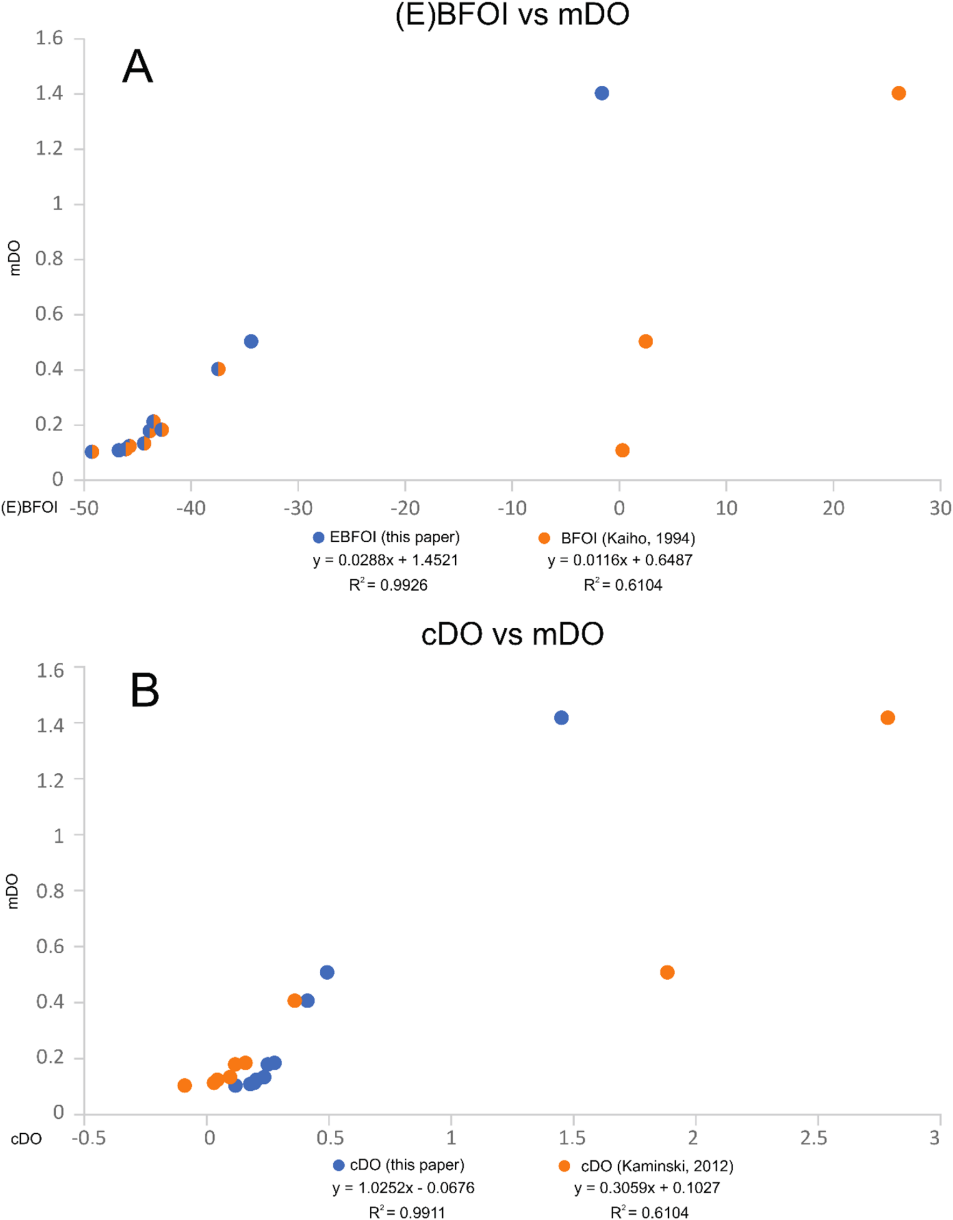
mDO vs. (E)BFOI and cDO (this paper and Kaminski). (**A**) Orange dots show the BFOI compared to the EBFOI (blue dots) plotted against the mDO by Schuhmacher^30^ suggesting a much better fit of the EBFOI (R^2^ = 0.9926) than the BFOI (R^2^ = 0.614) to mDO values. (**B**) Blue dots represent the cDO values using the EBFOI compared to the orange dots, representing the cDO values implicated by the linear regression after Kaiho^29^ providing the evidence of the better fit of the EBFOI compared to the BFOI to mDO values.

Data of Schuhmacher^30^ highlight the importance of integrating suboxic indicators to the equation and the use of Eq. (4) for samples yielding low abundant oxic indicators and Eq. (5) for calculating the whole livable habitat. We compare the BFOI and EBFOI values to the mDO by Schuhmacher^30^ (Fig. 5A). This shows the overestimation of oxic conditions following the formula of Kaiho^29^ of three samples that indicate low oxic conditions with 1.5–3 ml/l instead of the measured dys- and suboxic conditions, resulting in an R^2^ value of only 0.6104. Contrary, our new formula shows precise calculations of the DO values with a high R^2^ value of 0.9926. Hence our calculations provide increased accuracy of 38.2% for this dataset. Also, comparing the mDO values to our calculated cDO values and the cDO values implied by the linear regression after Kaiho^29^ displays the higher reliability of our new transfer function to calculate DO values compared to the original hypothesis of Kaiho (Fig. 5B).

### New BFOI and DO reconstructions in the Cenozoic

The BFOI values (Ref.^29^, Eqs. 1–2) are compared to the results of the EBFOI formulas (Eqs. 3–5) and the converted DO values (Eq. 6) on a Pliocene dataset (50 samples) of Garcia-Gallardo^81^, a Miocene dataset (310 samples) derived of 52 wells of the Austrian Vienna Basin of Kranner^71^ and an Oligocene dataset (33 samples) of Rupp and Ćorić^82^, all results are given in the Supplementary Table S2. These datasets were chosen to show the potential of our new formulas for fossil datasets, which fit well with the detailed reconstructions of paleoecological conditions by the authors. Garcia-Gallardo^81^ describes the Pliocene sediments they investigated as generally low oxic. Rupp and Ćorić^82^ also describe generally low oxic conditions with reoccurring extreme oxygen crises and Kranner^71^ provides a dataset with variable oxygenation, from high oxic to suboxic. Hence, the total of 383 samples covers a wide range of oxygen contents in different time slices of the Cenozoic. Comparing BFOI and EBFOI values (Fig. S2) underlines the same trend as in recent datasets. However, the BFOI calculation is highly biased to overestimate oxic conditions due to the lack of suboxic indicators in Eq. (1) and is therefore not a reliable proxy for DO estimations of fossil datasets. Thus, these authors only use qualitative or descriptive methods to reconstruct paleo-oxygenation (see Fig. 2). Using our newly introduced formula to calculate DO values (Eq. 6) corresponds well to the authors' oxygen reconstructions.

## Discussion

Paleoenvironmental reconstructions based on fossil datasets heavily rely on modern analogs (proxies) and their correlation. As a proxy for BWO, Kaiho^29^ introduced the BFOI. Applying this proxy to hundreds of fossil samples of the Miocene showed major offsets to the qualitative interpretation of the data (Fig. 2). Therefore, we checked the validity of this proxy on the original data and seven other recent datasets and adjusted the original formulas as described above to improve the value of benthic foraminifera as a proxy for oxygen concentrations. Including agglutinated foraminifers (representing at least 1/3 of all genus-level foraminifer taxa) in the calculation enhanced the method by preventing a loss of information by arbitrarily excluding wall textures from the analysis. Further, this may allow calculations for sediments underneath the CCD extending the calculation of DO values to all parts of the ocean, from shallow lagoonal settings to the abyssal plain and extreme habitats like hypersaline marshes. Including suboxic indicators in the equation of Kaiho^29^ further prevents an overestimation of oxic conditions, as can be seen in Fig. S1.

Four of the datasets^29,30,33,77^ were associated with mDO values. Comparing these mDO values to our new cDO values shows the overall fit of benthic foraminifers as a DO proxy using our formulas with an R^2^ value of 0.6311 (Fig. 3). The remaining offset between the cDO values and mDO values may be the results of two important caveats present in the available data: (1) inadequate DO values due to the distance of oceanographic stations to the sampling locations and (2) mDO values only consider BWO and do not include PWO, whereas our cDO values combine BWO and PWO. Especially the difference in mDO to cDO on the dataset of Kaiho^29^ are due to shortcoming 1, showing the importance of proxy data not only for fossil datasets but also for studies in modern oceans. Our new formulas, especially the transfer function enabling to calculate DO values directly, make tracking dissolved oxygen values easier and more efficient. Due to the fast response of benthic organisms and the extremely slow mobility of benthic foraminifers, this method contributes to tracking changing oxygen and nutrient conditions as precursors to localized anoxia in modern world oceans, which garnered significant scientific interest over the last decades for tracking OMZs^3,7–11^. Therefore, our new transfer function provides a high sensitivity of the proxy to eutrophication and decreasing oxygen due to changes in the sediment/water interface directly influencing the abundance and association of benthic foraminifers and may represent a tool not only for describing but also for predicting OMZs. Testing our new formulas on the fossil datasets of the Pliocene, Miocene and Oligocene, improved the proxy reliability for reconstructing changes in oxygenation of the whole habitat of geological records and offers a better fit to the established interpretations of the authors based on species abundance. To optimize the reconstruction of oxygen contents of fossil datasets provides relevant information of past climate changes and the general ocean chemistry and may allow tracking changes leading to major extinction events. These assumptions still need to be tested further on datasets before and after such events (e.g., OAEs) to better understand the impacts of climate change and anthropogenic influence (e.g., wastewater-based eutrophication). Applying our transfer function to recent datasets provides DO values of the whole livable habitat and therefore, will better understand and predict changes in the general oceanic metabolic cycle. Especially the impact of anthropogenic input (e.g., wastewater) increases oxygen depletion by eutrophication and leads to an expansion of OMZs in shallow marine environments. Thus, benthic foraminifers will respond quickly to these changes and may even provide the opportunity to still react to these changes. Rapid actions may prevent the prevalence emission of greenhouse gases like methane, nitrous oxide and chlorofluorocarbons which were fixed within world oceans for decades. Different approaches of applying benthic foraminiferal data have been carried out since the last decades, including analyses of test morphology, diversity, carbon isotopes, I/Ca proxy and measuring pore area percentages. While test morphology and diversity analyses can directly contribute to a better understanding of how foraminifers should be separated into dys-, sub- and oxic groups, the isotope analyses, the I/Ca proxy and the pore area measurements provide additional data. These independent analyses should be additionally applied whenever possible but may prove to be difficult if preservation of foraminiferal tests is not ideal or if chemicals were used during sampling (e.g., drilling fluid).

Thus, our newly introduced formulas (ideally combined with independent multiproxy approaches^27–38,43,44^) provide a major improvement in tracking changes in DO values more efficiently, allowing to predict changes in the general oceanic metabolic cycle and in reconstructing oxygen values in deep geological times.

## Methods

We enhance the method of Kaiho^29^ by (1) combining BWO with PWO, (2) including agglutinated taxa, and (3) using a better assignment of oxygen requirements (e.g., small suboxic taxa). As discussed, these changes make the DO reconstructions available for the whole livable habitat to trace and potentially predict OMZs.

### Calculation of oxygen concentration

To thwart an over-representation of oxic indicators, inevitably leading to an overestimated BFOI value, we integrate suboxic indicators into the original formula used for samples yielding oxic indicators (Eq. 1). However, adding the number of suboxic indicators into the formula would not result in the desired results. Doing so would suggest that suboxic indicators have the same importance as dysoxic and oxic indicators as suboxic taxa, even tough they can withstand high and low oxygen conditions^50,83^ and are present in oxic to dysoxic environments. Consequently, we factor them into the formula with only half the influence of oxic and dysoxic indicators. Kaiho^29^ also implemented this by splitting the suboxic indicators into groups (suboxics rather associated with low oxic conditions and suboxics rather associated with dysoxic conditions). We consequently enhance the original formula by adding ($\frac{S}{2}$) whereby "S" represents the abundance of suboxic indicators. To calculate not only the BWO but also the oxygenation of the whole livable habitat (BWO + PWO), we propose to use Eq. (3) only, if oxic indicators account for at least 10% of the whole fauna, due to the possibility of low abundant oxic species in low oxic conditions (see Fig. 1).

We agree with the formula of Kaiho^29^ used for calculating the DO values for samples lacking oxic indicators (Eq. 2). The only drawback of the formula arises with the assumption that oxic indicators always result in oxic conditions (see Fig. S1). Rather, we suggest adding the oxic indicators into the equation and using it also for samples yielding low (monadic) percentages of oxic indicators. The integration of oxic indicators does not effect the calculation if no oxic indicators are present. It consequently agrees with the original formula of Kaiho^29^ while providing much more realistic values with the mentioned low abundant oxic foraminifers (see Fig. 5).

However, these formulas cover the upper and lower end of the spectrum but do not describe the whole habitat where foraminifers dwell. Large parts of the fauna live shallow or deep infaunal^83,84^. Oxygen conditions deplete considerably by an increase in sediment depth. If samples yield more dysoxic than oxic indicators and oxic indicators account for at least 10% of the fauna (otherwise, Eq. (4) should be used), we suggest using a combination of both formulas (Eqs. 3, 4) to get the mean oxygenation of the whole livable habitat. By calculating the arithmetic mean of Eqs. (3) and (4), we derive Eq. (5) but adapt it further compared to Eq. (4). By integrating the oxic indicators only with their half impact ($\frac{O}{2}$) on the fauna, we prevent an overestimation that would otherwise occur if we added the oxic indicators without any adjustment. Using Eq. (5) to calculate the mean oxygenation of the whole habitat proved to be particularly useful to get realistic oxygen values of samples derived from low oxic to suboxic settings (see Fig. S1).

**Figure 6:**
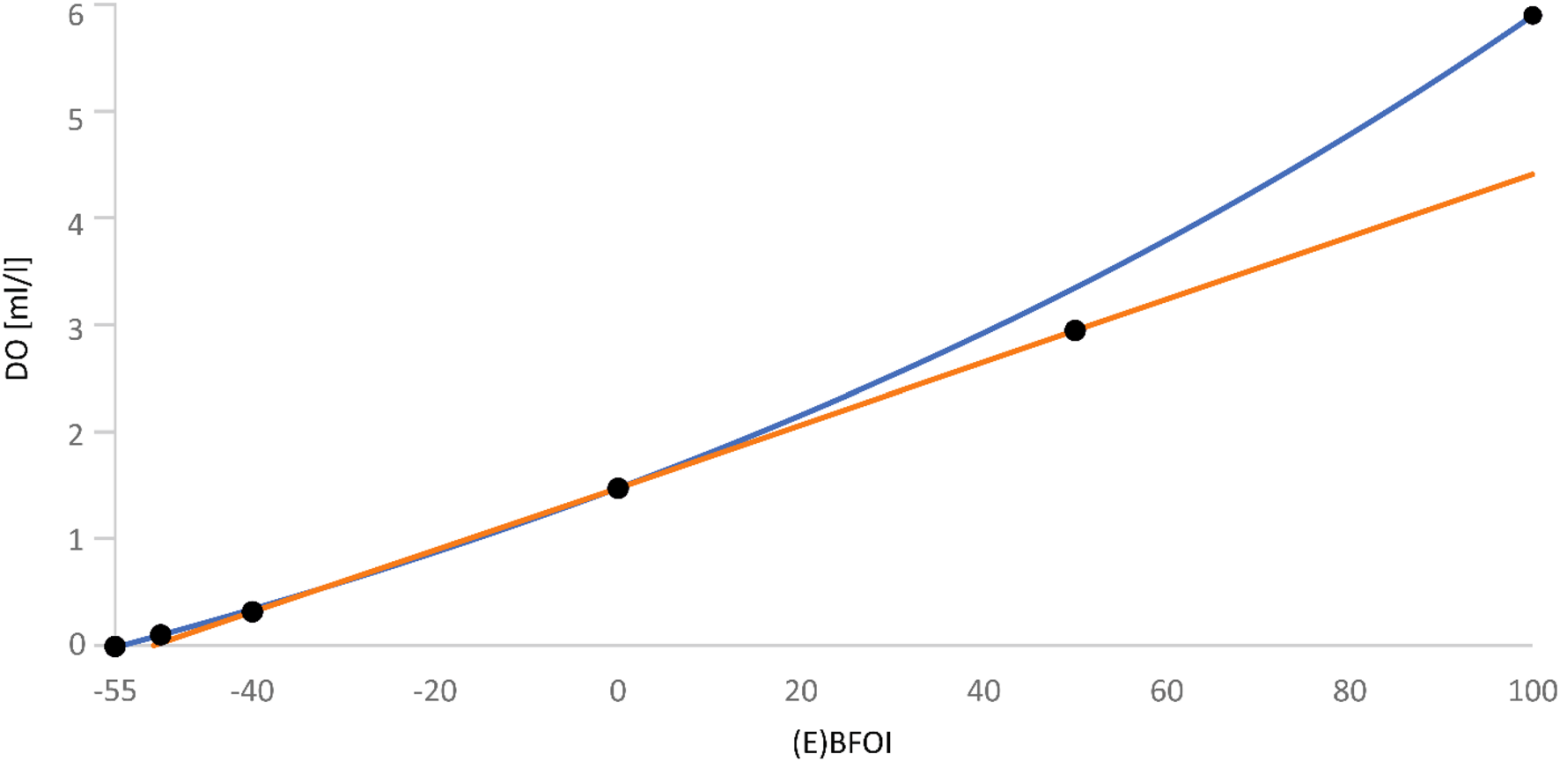
Relation of (E)BFOI and DO. Plotting the fixed (E)BFOI to dissolved oxygen (DO) values (see Table 1) as black dots in a cross plot. The orange line represents the linear regression trough the points (− 40/0.3), (0/1.5) and (50/3) whereas the blue line represents the best fit exponential function introduced by us based on using the fixed points (− 50/0.1), (0/1.5) and (100/6).

Further, we directly calculate the DO values [ml/l] with the exponential function (Eq. 6) we propose for the relation of (E)BFOI to DO. Our assumption that oxic conditions follow an exponential function rather than a linear function is based on the general behavior of oxygen, roughly following an exponential function while depleting^85^. Another indicator was plotting the fixed points introduced by Kaiho^29^ in a cross plot (Fig. 6). Already with the first description of Kaiho^29^, an attempt was made to correlate the BFOI values to DO values. He suggested a nearly linear relation between BFOI and DO values. The relation, postulated by Kaiho^29^, is shown in Table 1. This implements a fixed BFOI value correlation to DO values after Kaiho^29^ like this: 100 BFOI = 6.0 [ml/l]; 50 BFOI = 3.0 [ml/l]; 0 BFOI = 1.5 [ml/l]; − 40 BFOI = 0.3 [ml/l] and − 50 = 0.1 [ml/l]. Plotting these fixed points in a cross plot (Fig. 6_black dots) shows that only three points can be linked linearly and no overall linear regression (Fig. 6_orange) of DO to BFOI can be assumed. Much more likely is an exponential relation. Best approximation of the exponential formula (Fig. 6_blue) is given by using the fixed points (100 BFOI/6 [ml/l]), (0 BFOI/1.5 [ml/l]) and (− 50 BFOI/0.1 [ml/l]). Furthermore, Kaminski^33^ already stated that only three of the five fixed values follow a linear regression but did not introduce a new way of correlation and used visual correlation with a physical ruler to estimate the relation of BFOI to DO after Kaiho^29^.

### Calibrations with recent datasets

To validate the EBFOI formulas, we used the original data of Kaiho^29^ as well as that of Piller and Haunold^76^, Schuhmacher^30^, Kaminski^33^, Amao^77^, Charrieau^78,79^, and Groenevelt^80^ originating from different oceans. To calculate the EBFOI values, we used the classification of benthic foraminifers as oxic, suboxic and dysoxic given in the corresponding original publications. However, in contrast to those authors, we assessed small-sized oxic indicators, not as suboxic class A^29,33^ but added them to the oxic indicators due to the possibility that other ecological parameters can influence foraminiferal growth^83,86–89^.

Further, we tested our new transfer function on all datasets providing mDO values, including the original data of Kaiho^29^ and those of Schuhmacher^30^, Kaminski (2012) and Amao^77^, covering different oxygen concentrations from low oxic to high oxic (see Table S1), as described below.

**Figure 7:**
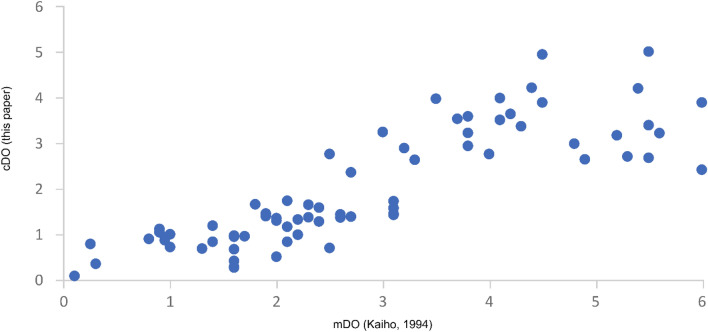
mDO vs cDO of the dataset of Kaiho^29^. Cross plot comparing the mDO by Kaiho^29^ and cDO values of this paper calculated by Eq. (6).

Calculating DO concentrations with our new formulas (Eqs. 3–6) for the original data of Kaiho^29^ give diverging values (Fig. 7). This might be a side effect of measuring and extrapolating water column data (mDO) of the nearest available oceanographic station to the sampling position and the poor resolution. Therefore, assumed mDO values can be misleading. Generally, the calculation of the cDO (Eq. 6) using EBFOI values (Eqs. 3–5) shows an extremely good fit to about 1/3 of the samples (low oxic and high oxic). We realized that all samples of the North Atlantic show a misfit of 2.1–3.6 [ml/l], leading to the conclusion that there might be a rather big offset regarding the stations measuring the DO values compared to the real DO at the sampling location. We also see smaller misfits from mDO to our cDO for other samples, which cannot easily be explained by loosely correlating mDO over distance to the sampling locations. More likely, these offsets are explainable by the depletion of oxygen in the sediment and our calculation of oxygen values of the whole livable habitat, not only the BWO. Especially, samples yielding high numbers of suboxic indicators show a large offset from mDO to our cDO values (see Fig. S1). Sample V18-186 of the Indian Ocean, for example, contains more than 75% suboxic indicators. According to the mDO of Kaiho^29^ this sample shows a BWO of 4.9 [ml/l] but our calculations only gives a value of 2.7 [ml/l]. The high amount of suboxic indicators reflect mostly species living infaunal^29,84^.

Consequently, it seems likely that the BWO above the seafloor might be considerably higher than the PWO. Our aim to calculate the whole livable habitat can therefore result in these offsets. Nevertheless, the trends of all analyzed samples are similar, and using our new formulas provides reliable estimations of DO values.

The dataset of Schuhmacher^30^ was derived from studies of the OMZ of the continental margin in the Arabian Sea. The BFOI of most of these samples can be calculated by the formula of Kaiho (O = 0, Eq. 2). Nevertheless, some samples yield few oxic indicators, where we would have to apply the formula $\mathrm{O}>0$ (Eq. 1), leading to at least low oxic conditions and untypically high DO values. Using our enhanced formulas (Eqs. 4, 5) resulted in much more realistic values for samples CD145_55808#3, CD146_55901#11, CD145_55830#3 and CD145_55818#4 (see Fig. 5). The DO values calculated by Eq. (6) using the EBFOI results (Eqs. 4, 5) are very close to the measured DO values given by Schuhmacher^30^. Especially the four mentioned samples show an excellent correlation to the measured DO values. For three samples (CD145_55808#3, CD146_55901#11, CD145_55830#3) the cDO values are identical to the mDO values and the fourth (CD145_55818#4) is only slightly too high (~ 0.07 ml/l).

Calculating the DO values with our new formula (and EBFOI formulas) (see Fig. 4) for the dataset of Kaminski^33^ shows much less aberration to the mDO values than within the dataset of Kaiho^29^, underlining our interpretation that the misfits of the North Pacific samples of Kaiho most likely reflect unreliably measured and extrapolated DO values. The differences and sometimes good fit of the cDO values of Kaminski^33^ using a physical ruler to linearly convert BFOI to DO values result in differences in the calculated (E)BFOI values. Nevertheless, our new cDO values show a better mean fit to the mDO values than the cDO values of Kaminski^33^. Similar to the smaller offsets in the dataset of Kaiho^29^, we explain the offsets from our cDO to the mDO values with our formulas aiming for total DO values and not only the BWO values.

### Oxygen calculations on fossil datasets

The Oligocene dataset of Rupp and Ćorić^72^ shows generally big differences in BFOI (mean of 33) and EBFOI (mean of 8; see Fig. S2). EBFOI calculations are considerably lower all over the analyzed samples, resulting in relatively low cDO (using Eq. 6) values. The low cDO values fit the reconstruction of Rupp and Ćorić^72^, who describe repeated oxygen crises and generally low oxic conditions for both localities for the represented timeframes.

The Miocene dataset of Kranner^71^ shows that the EBFOI calculations correlate much better to the qualitative oxygen interpretations based on benthic foraminifers presented in Kranner^71^. In contrast, the BFOI displayed a major overestimation of high oxic conditions (see Fig. 2). The huge differences of the BFOI and EBFOI can be seen in the supplementary (Fig. S2, Table S2). Thus, the EBFOI calculations and the concomitant calculations of cDO values fit much better to the reconstructed paleoenvironments of Kranner^71^.

The Pliocene dataset of Garcia-Gallardo^81^ also shows a major offset between the BFOI^29^ and EBFOI calculations. The mean BFOI for all samples is 38, whereas the mean EBFOI is only 12 (see Table S2). Therefore, our cDO values indicate generally low oxic to partly suboxic conditions, fitting to Garcia-Gallardo's general reconstruction of the investigated area. Thus, a well-suitable correlation for fossil datasets is shown on these three examples, using the EBFOI and the cDO formulas, providing a major improvement in reconstructing oxygen values of geological times using benthic foraminifers as a proxy.

## Supplementary Information

Supplementary Information 1.

Supplementary Information 2.

Supplementary Information 3.

Supplementary Information 4.

Supplementary Information 5.
