# Supplementary material for: Calculating dissolved marine oxygen values based on an enhanced Benthic Foraminifera Oxygen Index
Source: Sci Rep. 2022 Jan 26;12:1376. doi: 10.1038/s41598-022-05295-8 (PMC8791969; doi:10.1038/s41598-022-05295-8)
Supplement: Supplementary file 3 — Supplementary Information 3. [file 41598_2022_5295_MOESM3_ESM.pdf]

Supplementary of "Calculating dissolved marine oxygen values based on a revised Benthic Foraminifera Oxygen Index"

M. Kranner[1]*, M. Harzhauser[1], C. Beer[3], G. Auer[2], W.E. Piller[2]

[1]Geological-Palaeontological Department, Natural History Museum Vienna, Burgring 7, 1010 Vienna, Austria
[2]Institute of Earth Sciences (Geology and Palaeontology), NAWI Graz Geocenter, University of Graz, Heinrichstr. 26, 8010 Graz, Austria
[3]PwC Austria, Donau-City-Straße 7, 122 Vienna, Austria

*Corresponding author: matthias.kranner@nhm-wien.ac.at; phone +43 1 52177 255

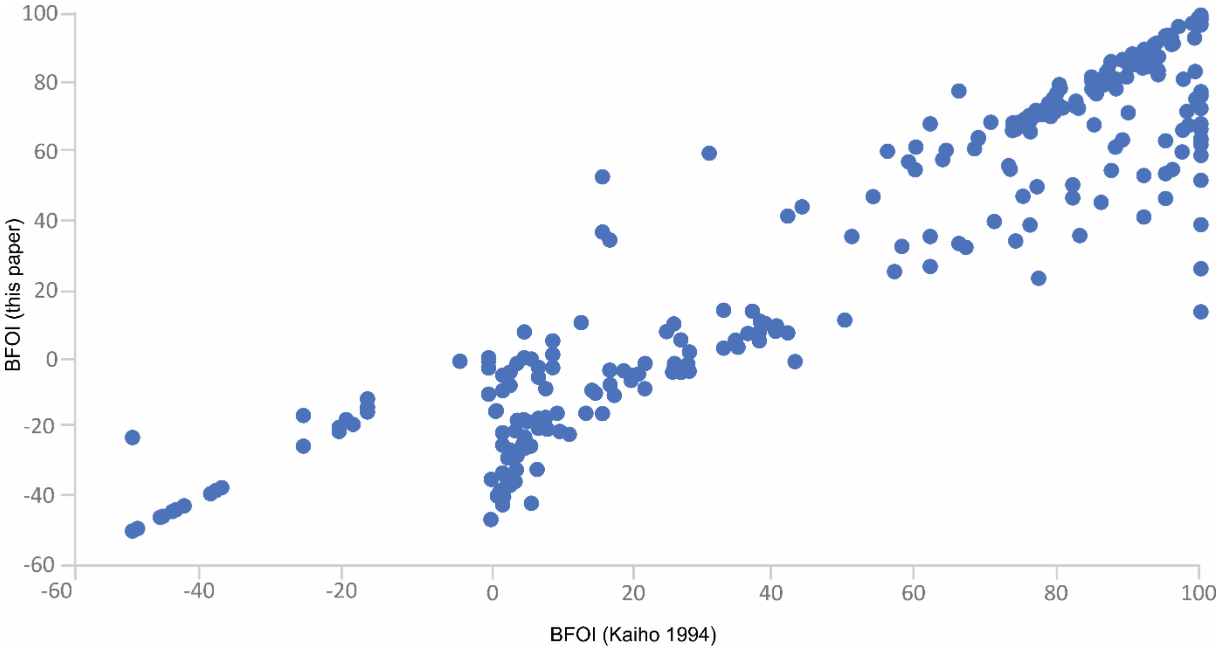

Figure S1. BFOI (Kaiho) vs. BFOI (this paper) of all recent datasets. Comparing old BFOI values after Kaiho (29) and BFOI values of this paper resulting of equation 3–5 of all eight recent datasets (29, 30, 33, 76–80).

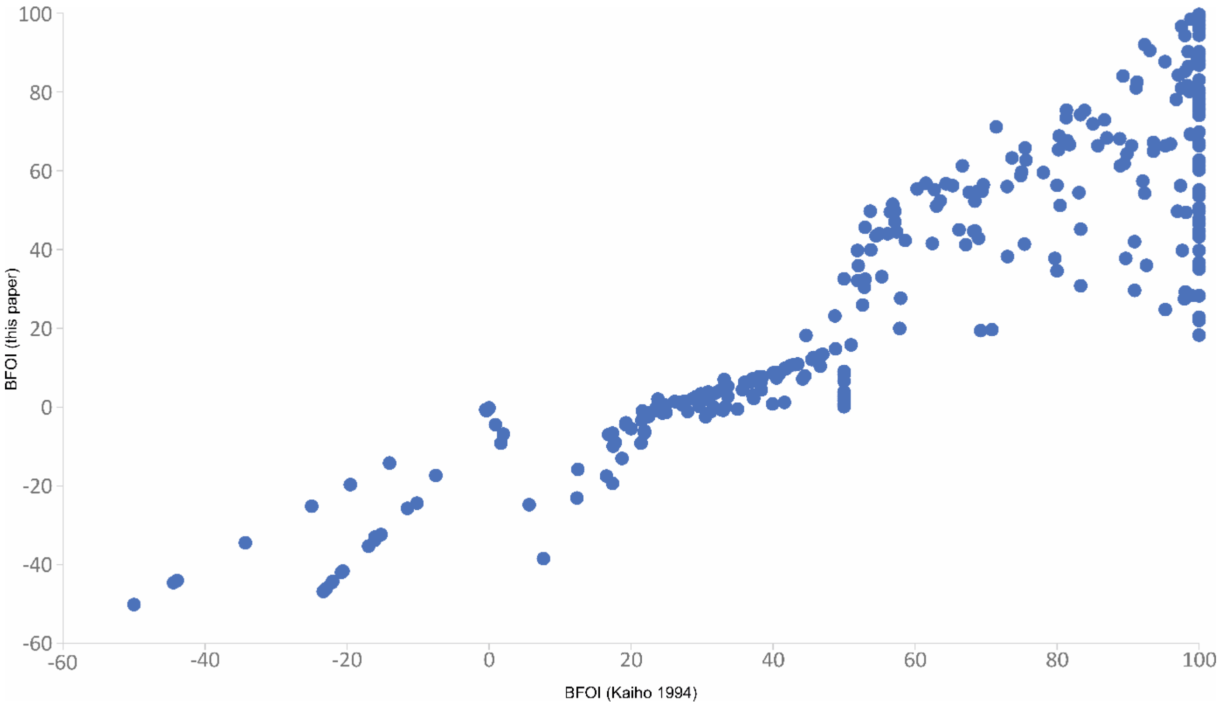

Figure S2. BFOI (Kaiho) vs. BFOI (this paper) of all fossil datasets Comparing old BFOI values after Kaiho (29) and BFOI values of this paper resulting of equation 3–5 of all three fossil datasets (71, 81, 82).

Table S1. All 275 recent samples with the old BFOI values after Kaiho (29), comparing BFOI values of this paper (Eq. 3–5) and our cDO values derived from the transfer function (Eq. 6). 142 of these samples also provide mDO values measured by the authors (29, 30, 33, 77). Seventy-two (72) samples were investigated by Kaiho (29); the mDO values [ml/l] were measured at the nearest oceanic station of each sampling location. Eleven (11) samples were investigated by Schuhmacher (30) out of the oxygen minimum zone of the Arabian Sea. Thirty (30) samples were investigated by Kaminski (33) of the Marmara Sea to calibrate the BFOI formula in the Mediterranean; Kaminski also estimated dissolved oxygen (cDO, Kaminski) determined by using a physical ruler to linearly correlate with the BFOI-DO relation introduced by Kaiho (29). Twentynine (29) samples have been investigated by Amao (77) of the Persian Gulf. The other 133 samples do not provide mDO values but at least a descriptive oxygen estimation. Seventy-three (73) samples were investigated by Piller and Haunold (76) of the Safaga Bay (Red Sea). Sixty (60) samples derived from the Baltic Sea; 22 samples were investigated by Charrieu (79), whereas 32 samples were investigated by Charrieu (78) and six samples of Groenevelt (80), including living and dead assemblages.

Table S2. All 393 analyzed fossil samples with the calculated BFOI values after Kaiho (29), comparing our newly calculated BFOI values (Eq. 3–5), as well as our calculated dissolved oxygen (cDO) values [ml/l] (Eq. 6). Fifty (50) Pliocene samples derived from the Gulf of Cadiz, investigated by Garcia-Gallardo (81), 310 Miocene core samples in stratigraphic order, investigated by Kranner (71) of 52 wells of the Austrian Vienna Basin and 33 Oligocene samples investigated by Rupp and Ćorić (72) of two locations in Lower Austria (Unterrudling, UR and Polsenz, PO).

Table S3. List of 418 species level taxa of recent and fossil benthic foraminifers with their associated oxygenation preferences (O=oxic, S=suboxic, D=dysoxic) compiled of literature [e.g.: Kaiho (29),

Schuhmacher (30), Kaminski (33), Kranner (71), Jorissen (54), Ohkushi (36), Moffit (42), Palmer (40) and Tetard (41) and references within].
